# Expression of nestin in cancer: a tissue microarray study involving 13,318 cancers from 148 tumor entities

**DOI:** 10.1186/s12885-026-17190-w

**Published:** 2026-11-02

**Authors:** Nina Schraps, Muriel Torzewski, Clara von Bargen, Fiete Gehrisch, Anne Menz, Florian Lutz, Viktoria Chirico, Florian Viehweger, David Dum, Ria Schlichter, Andrea Hinsch, Christoph Fraune, Christian Bernreuther, Seyma Büyücek, Martina Kluth, Claudia Hube-Magg, Georgia Makrypidi-Fraune, Katharina Möller, Andreas M Luebke, Patrick Lebok, Guido Sauter, Maximilian Lennartz, Till Clauditz, Andreas H Marx, Thilo Hackert, Ronald Simon, Eike Burandt, Natalia Gorbokon, Maria Christina Tsourlakis, Sarah Minner, Till Krech, Morton Freytag, Viktor Reiswich, Stefan Steurer

**Affiliations:** 1 Institute of Pathology, University Medical Center Hamburg-Eppendorf, Martinistrasse 52, 20246 Hamburg, Germany; 2 General, Visceral and Thoracic Surgery Department and Clinic, University Medical Center Hamburg-Eppendorf, Hamburg, Germany; 3 Institute of Pathology, Clinical Center Osnabrueck, Osnabrueck, Germany; 4 Department of Pathology, Academic Hospital Fuerth, Fuerth, Germany

**Keywords:** Nestin, Tissue microarray, Immunohistochemistry, Human cancer

## Abstract

**Background:**

Nestin (neuroepithelial stem cell protein) is a class VI intermediate filament protein which is transiently expressed in subsets of precursor cells of various tissues during development. Nestin expression can be reinduced in the adult during pathological situations.

**Methods:**

To better comprehend the prevalence of nestin in different cancer types, nestin was analyzed by immunohistochemistry on tissue microarrays (TMAs) containing 17,285 samples from 148 different tumor types. Our antibody was validated using a second independent antibody on 76 different normal tissues, ensuring a broad range of proteins to be tested for cross-reactivity.

**Results:**

Of 13,318 analyzable tumors, nestin staining was absent in 11,677 (87.7%), weak in 961 (7.2%), moderate in 360 (2.7%), and strong in 320 (2.4%) cases. Of 148 tumor categories, 119 showed nestin staining in at least one case, and 57 included at least one sample with strong positivity. Nestin positivity was most seen in tumors of the salivary glands (up to 100%), gastrointestinal stromal tumors (GIST) (93.8%), tumors of the peripheral nerves (up to 93.8%) and malignant melanomas (81.8%). High nestin expression was linked to high grade (*p* < 0.0001), estrogen (*p* = 0.0054), and progesterone receptor negativity (*p* = 0.0051), as well as a triple negative phenotype (*p* = 0.0028) in invasive breast cancer NST, in papillary as well as poor ISUP (p=0.0149), Fuhrman (p=0.0006) and Thoenes grades (p=0.0099) in clear cell renal cell carcinoma, invasive tumor growth in urothelial carcinoma of the urinary bladder (pTa vs. pT2-4; *p* < 0.0001), and advanced pT stage (*p* = 0.0477) in serous carcinoma of the ovary.

**Conclusions:**

The data from this study provide an overview of nestin expression in human cancer and demonstrate that increased nestin levels can occur in many different tumor entities. Significant associations with unfavorable tumor characteristics in multiple entities argue for a role of nestin in driving cancer aggressiveness, which may further support the concept of exploring nestin as a drug target.

**Supplementary information:**

The online version contains supplementary material available at https://doi.org/10.1186/s12885-026-17190-w.

## Introduction

Nestin (*ne*uroepithelial *st*em cell prote*in*) is a class VI intermediate filament protein which preferentially co-assembles with vimentin or internexin-alpha to form heterodimer coiled-coil molecules supporting the cellular architecture [1,2]. It is transiently expressed in subsets of precursor cells of the peripheral and the central nervous system, muscle and other tissues during development [1]. Upon differentiation, nestin expression becomes replaced by tissue-specific intermediate filament proteins in most cell types [1]. Nestin expression is reinduced in cells of the adult during pathological situations, such as the formation of the glial scar after central nervous system injury and during regeneration of injured muscle tissue [3–5]. Nestin expression is widely used as a marker for central nervous system progenitor cells and was also proposed to serve as a marker for detecting newly formed (proliferative) endothelial cells [1,2,6]. Nestin also has relevant roles in cancer biology. Nestin was shown to promote tumor cell proliferation, migration, and invasion in cell lines derived from glioblastoma [7,8], breast [9,10], lung [11], colorectal [12], and pancreatic cancers [13]. In xenograft models, nestin overexpression in tumor cells enhanced tumor growth [14, 15], invasion [7,13,16] and vascularization [17–19] while nestin inhibition slowed tumor progression and impaired angiogenesis [7,14,17]. Expression of nestin has been described to correlate with unfavorable tumor features and/or poor patient prognosis in melanoma [20–22], lung [23], breast [24–27], and pancreatic cancer [28]. Nestin expression was also found to be related to poor response to radiotherapy in glioblastoma [29] and chemotherapy in hepatocellular carcinoma (HCC) [30], lung [31] and esophageal [32] cancer. However, published data on the prevalence of nestin expression in tumor cells are quite variable and range, for example, from 25.0 to 100.0% in malignant melanoma [33,34], 18.8–100% of squamous cell carcinomas of the skin [34,35], 9.9–88.7% in pulmonary adenocarcinoma [23,36], 11.7–70.5% in invasive breast cancer NST [37,38] and 32.5–95.5% of serous ovarian cancers [39–41]. These divergent findings are likely to be due to the use of different antibodies, staining protocols, and criteria for defining nestin positivity.

Considering these promising data on a role of nestin in cancer, the variability of data in tumor entities that were earlier examined and the lack of data for many other important tumor entities, an extensive and highly standardized survey of nestin expression in a broad range of different tumor types is needed. Nestin expression was thus evaluated in more than 17,000 tumor tissue samples from 148 different tumor types and subtypes as well as 76 different non-neoplastic tissue types by immunohistochemistry (IHC) in a tissue microarray (TMA) format in this study.

## Materials and methods

### Tissue microarrays (TMAs)

Our normal tissue TMA was composed of 8 samples from 8 different donors for each of 76 different normal tissue types (608 samples on one slide). The cancer TMAs contained a total of 17,285 primary tumors from 148 tumor types and subtypes. Detailed histopathological and molecular data were available for cancers of the breast (n = 1300), kidney (n = 2,456), bladder (n = 2,140), pancreas (n = 598), stomach (n = 327), endometrium (n = 182), ovary (n = 369), and the colorectum (n = 2,351). The composition of both normal and cancer TMAs is described in detail in the results section. All samples were from the archives of the Institutes of Pathology, University Hospital of Hamburg, Germany, the Institute of Pathology, Clinical Center Osnabrueck, Germany, and Department of Pathology, Academic Hospital Fuerth, Germany. Tissues were fixed in 4% buffered formalin and then embedded in paraffin. The TMA manufacturing process was described earlier in detail [42,43]. TMA tissue spot diameter was 0.6 mm. The use of archived remnants of diagnostic tissues for manufacturing of TMAs and their analysis for research purposes as well as patient data analysis has been approved by local laws (HmbKHG, § 12) and by the local ethics committee (Ethics commission Hamburg, WF-049/09). All work has been carried out in compliance with the Helsinki Declaration

### Immunohistochemistry

Freshly cut TMA sections were immunostained on one day and in one experiment. Slides were deparaffinized with xylol, rehydrated through a graded alcohol series and exposed to heat-induced antigen retrieval for 5 min in an autoclave at 121 °C in pH 7.8 Tris-EDTA-Citrat (TEC) puffer. Endogenous peroxidase activity was blocked with Dako REAL Peroxidase-Blocking Solution (Agilent Technologies, Santa Clara, CA, USA; #S2023) for 10 min. Primary antibody specific for nestin (recombinant mouse monoclonal antibody, ARX-604, MS Validated Antibodies GmbH, Hamburg, Germany) was applied at 37 °C for 60 min at a dilution of 1:100. For the purpose of antibody validation [44], the normal tissue TMA was also analyzed by the rabbit recombinant monoclonal nestin antibody (clone-ID SP103, Abcam, Cambridge, UK) at a dilution of 1:15 and an otherwise identical protocol. Bound antibody was then visualized using the Dako REAL EnVision Detection System Peroxidase/DAB+, Rabbit/Mouse kit (Agilent Technologies, Santa Clara, CA, USA; #K5007) according to the manufacturer’s directions. The sections were counterstained with hemalaun. IHC scoring was predefined and has been used in multiple previous studies [45–47]. For tumor tissues, the percentage of positive neoplastic cells was estimated, and the staining intensity was semi-quantitatively recorded (0, 1+, 2+, 3+) [48]. In addition, it was recorded whether the staining involved membranes, cytoplasm, and or nuclei. For statistical analyses, the staining results were categorized into four groups. Tumors without any staining were considered negative. Tumors with 1 + staining intensity in ≤ 70% of tumor cells or 2 + intensity in ≤ 30% of tumor cells were considered weakly positive. Tumors with 1 + staining intensity in > 70% of tumor cells, 2 + intensity in 31–70%, or 3 + intensity in ≤ 30% of tumor cells were considered moderately positive. Tumors with 2 + intensity in > 70% or 3 + intensity in > 30% of tumor cells were considered strongly positive

### Statistics

Statistical calculations were performed with JMP18^®^ software (SAS^®^, Cary, NC, USA). Contingency tables and the chi²-test were performed to search for associations between nestin immunostaining and tumor phenotype.

## Results

### Technical issues 

A total of 13,318 (77.0%) of 17,285 tumor samples were interpretable in our TMA analysis. Non-interpretable samples demonstrated lack of unequivocal tumor cells or a complete lack of tissue

### Nestin in normal tissues

**Fig. 1:**
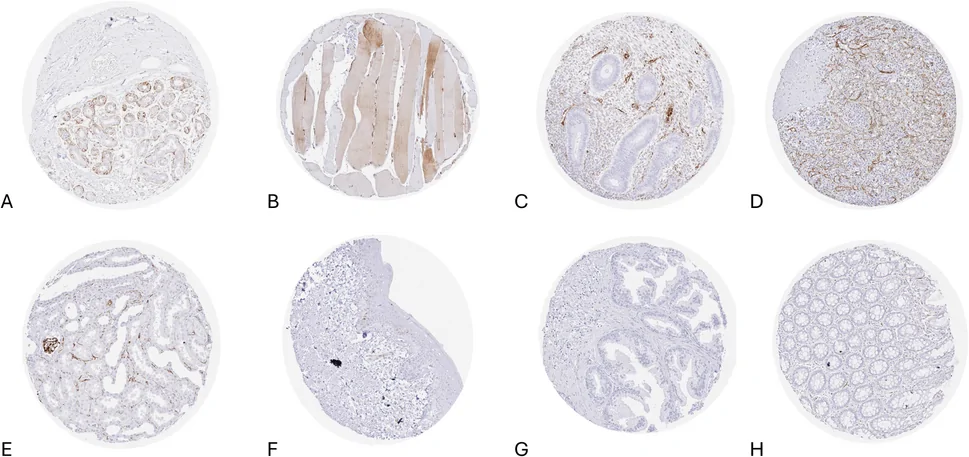
Nestin staining in normal tissues.The panels show a distinct predominantly membranous nestin staining of endothelial cells in all tissues. In the breast, additional strong membranous staining occurs in myoepithelial cells (**A**). In skeletal muscle (**B**) an additional cytoplasmic staining is prominent in muscle cells. In the endometrium, most stroma cells show a weak to moderate nestin staining (**C**). Nestin staining is limited to endothelial cells in the spleen (**D**), kidney (**E**), skin (**F**), prostate (**G**), and colon (**H**)

Nestin immunostaining predominated in endothelial cells where it was seen in all tissue types. It was most common in small capillaries of the heart. The intensity of nestin staining in endothelial cells displayed some variability between tissues from different donors. A strong nestin staining was also seen in myoepithelial cells of the breast and in some “altered” skeletal muscle cells with atrophic features. A weak to moderate nestin positivity occurred in endometrial stroma cells and in myometrium cells of some samples. Images of tissue nestin staining are given in Fig. 1. All nestin positive cell types detected by ARX-604 were also confirmed by using a second antibody SP103. For SP103 an additional membranous and/or cytoplasmic staining was seen in numerous further tissues, including for example renal tubular cells, prostatic and colonic epithelium (Supplementary Fig. 1)

### Nestin in cancer

**Fig. 2:**
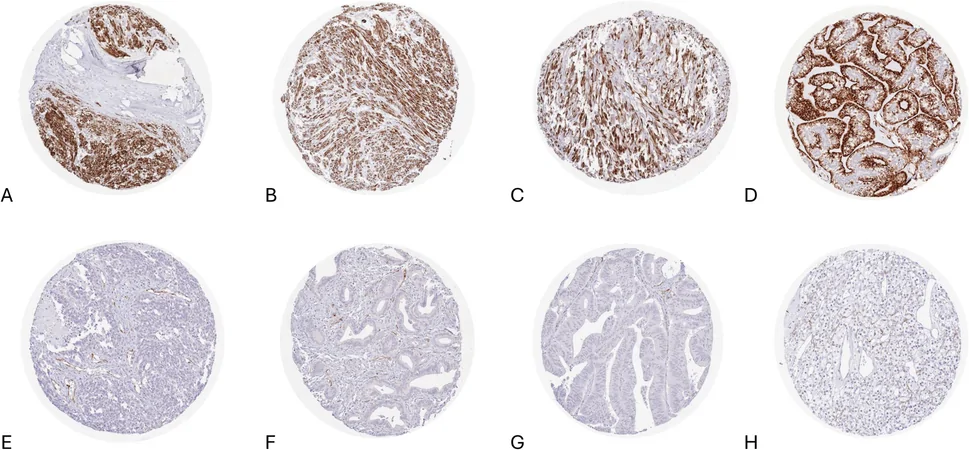
Nestin immunostaining in cancer. The panels show a distinct predominantly membranous but also cytoplasmatic nestin staining in a gastrointestinal stromal tumor (**A**), an angiosarcoma (**B**), a leiomyosarcoma (**C**), and an epithelial-myoepithelial carcinoma of the salivary gland (**D**), while nestin staining is limited to stromal cells in a serous carcinoma of the ovary (**E**), a ductal adenocarcinoma of the pancreas (**F**) an adenocarcinoma of the colon (**G**) and a clear cell renal cell carcinoma (**H**) lacking nestin staining in tumor cells

**Fig. 3:**
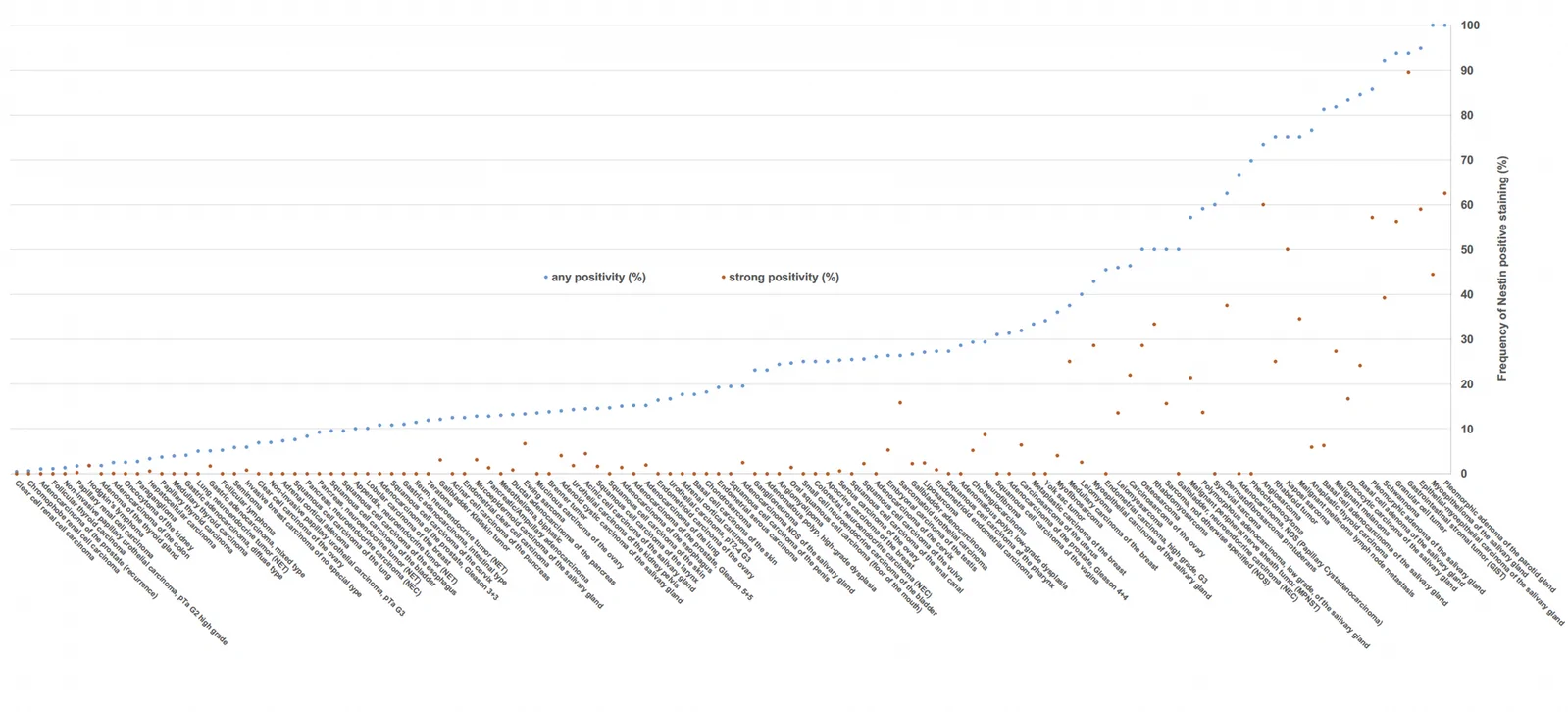
Ranking order of nestin positive immunostaining in different human tumors. Orange dots show the percentage of strongly stained samples, while blue dots show the percentage of positive samples of any intensity

Among 13,318 analyzable tumors, nestin staining was absent in 11,677 cases (87.7%). It was considered weak in 961 (7.2%), moderate in 360 (2.7%), and strong in 320 (2.4%) cases. Of 148 tumor categories, 119 showed nestin expression in at least one case, and 57 included at least one sample with strong nestin positivity (Table 1). Nestin positivity was most seen in tumors of the salivary glands (up to 100%), gastrointestinal stromal tumors (GIST) (93.8%), tumors of the peripheral nerves (up to 93.8%) and malignant melanomas (81.8%). Representative images of nestin staining of cancers are shown in Fig. 2 and Supplementary Fig. 2 A graphical representation of a ranking order of nestin positive tumors and of strongly positive tumors is given in Fig. 3. The relationship between nestin staining and histopathological or molecular features in various cancer types is shown in Table 2. High nestin expression was linked to high grade (p < 0.0001), ER (p = 0.0054), and PR negativity (p = 0.0051), as well as a triple negative phenotype (p = 0.0028) in invasive breast cancer NST, poor ISUP (p = 0.0101), and Fuhrman grades (p = 0.0230) in papillary renal cell carcinoma, poor ISUP (p = 0.0149), Fuhrman (p = 0.0006) and Thoenes grades (p = 0.0099) in clear cell renal cell carcinoma (RCC), invasive tumor growth in urothelial carcinoma of the urinary bladder (pTa vs. pT2-4; p < 0.0001), and advanced pT Stage (p = 0.0477) in serous carcinoma of the ovary. The nestin expression level was unrelated to features of cancer aggressiveness in pancreatic, gastric, endometrial and colorectal cancer.

**Table 1:** Nestin immunostaining in tumors

Tumor category				Nestin immunostaining			
	Tumor entity	on TMA (*n*)	analyzable (*n*)	negative (%)	weak (%)	moderate (%)	strong (%)
Tumors of the skin	Basal cell carcinoma of the skin	41	17	82.4	17.6	0.0	0.0
	Squamous cell carcinoma of the skin	95	75	85.3	14.7	0.0	0.0
	Malignant melanoma	19	11	18.2	18.2	36.4	27.3
	Malignant melanoma lymph node metastasis	86	84	25.0	28.6	11.9	34.5
	Merkel cell carcinoma	2	1	100.0	0.0	0.0	0.0
Tumors of the head and neck	Squamous cell carcinoma of the larynx	109	62	85.5	11.3	1.6	1.6
	Squamous cell carcinoma of the pharynx	60	44	72.7	13.6	13.6	0.0
	Oral squamous cell carcinoma (floor of the mouth)	130	73	75.3	21.9	1.4	1.4
	Pleomorphic adenoma of the parotid gland	50	24	0.0	29.2	8.3	62.5
	Warthin tumor of the parotid gland	104	68	100.0	0.0	0.0	0.0
	Adenocarcinoma, NOS (Papillary Cystadenocarcinoma)	14	3	33.3	66.7	0.0	0.0
	Salivary duct carcinoma	15	8	100.0	0.0	0.0	0.0
	Acinic cell carcinoma of the salivary gland	181	90	85.6	7.8	2.2	4.4
	Adenocarcinoma NOS of the salivary gland	109	41	80.5	9.8	7.3	2.4
	Adenoid cystic carcinoma of the salivary gland	180	50	86.0	4.0	6.0	4.0
	Basal cell adenocarcinoma of the salivary gland	25	16	18.8	56.3	18.8	6.3
	Basal cell adenoma of the salivary gland	101	58	15.5	34.5	25.9	24.1
	Epithelial-myoepithelial carcinoma of the salivary gland	53	39	5.1	20.5	15.4	59.0
	Mucoepidermoid carcinoma of the salivary gland	343	195	87.2	6.2	3.6	3.1
	Myoepithelial carcinoma of the salivary gland	21	7	57.1	14.3	0.0	28.6
	Myoepithelioma of the salivary gland	11	9	0.0	33.3	22.2	44.4
	Oncocytic carcinoma of the salivary gland	12	6	16.7	16.7	50.0	16.7
	Polymorphous adenocarcinoma, low grade, of the salivary gland	41	22	40.9	36.4	9.1	13.6
	Pleomorphic adenoma of the salivary gland	53	14	14.3	14.3	14.3	57.1
Tumors of the lung, pleura and thymus	Adenocarcinoma of the lung	196	158	84.8	13.3	0.0	1.9
	Squamous cell carcinoma of the lung	80	66	92.4	7.6	0.0	0.0
	Mesothelioma, epithelioid	32	28	100.0	0.0	0.0	0.0
	Mesothelioma, biphasic	25	23	87.0	4.3	8.7	0.0
	Thymoma	29	18	100.0	0.0	0.0	0.0
	Lung, neuroendocrine tumor (NET)	29	20	95.0	5.0	0.0	0.0
Tumors of the female genital tract	Squamous cell carcinoma of the vagina	30	29	69.0	31.0	0.0	0.0
	Squamous cell carcinoma of the vulva	107	90	74.4	20.0	3.3	2.2
	Squamous cell carcinoma of the cervix	88	83	89.2	10.8	0.0	0.0
	Adenocarcinoma of the cervix	23	23	73.9	17.4	8.7	0.0
	Endometrioid endometrial carcinoma	288	242	72.7	22.7	3.7	0.8
	Endometrial serous carcinoma	36	26	80.8	15.4	3.8	0.0
	Carcinosarcoma of the uterus	57	47	68.1	21.3	4.3	6.4
	Endometrial carcinoma, high grade, G3	13	11	54.5	27.3	18.2	0.0
	Endometrial clear cell carcinoma	9	8	87.5	12.5	0.0	0.0
	Endometrioid carcinoma of the ovary	93	55	83.6	12.7	3.6	0.0
	Serous carcinoma of the ovary	530	336	74.7	22.9	1.8	0.6
	Mucinous carcinoma of the ovary	75	37	86.5	10.8	2.7	0.0
	Clear cell carcinoma of the ovary	51	29	93.1	6.9	0.0	0.0
	Carcinosarcoma of the ovary	47	41	53.7	22.0	2.4	22.0
	Granulosa cell tumor of the ovary	44	42	100.0	0.0	0.0	0.0
	Leydig cell tumor of the ovary	4	4	100.0	0.0	0.0	0.0
	Sertoli cell tumor of the ovary	1	1	100.0	0.0	0.0	0.0
	Sertoli Leydig cell tumor of the ovary	3	2	100.0	0.0	0.0	0.0
	Steroid cell tumor of the ovary	3	3	100.0	0.0	0.0	0.0
	Brenner tumor	32	29	86.2	3.4	10.3	0.0
Tumors of the breast	Invasive breast carcinoma of no special type	1396	1186	94.1	3.9	1.3	0.8
	Lobular carcinoma of the breast	159	129	89.9	7.0	3.1	0.0
	Medullary carcinoma of the breast	8	8	62.5	12.5	0.0	25.0
	Tubular carcinoma of the breast	20	19	100.0	0.0	0.0	0.0
	Mucinous carcinoma of the breast	18	12	100.0	0.0	0.0	0.0
	Apocrine carcinoma of the breast	4	4	75.0	25.0	0.0	0.0
	Metaplastic carcinoma of the breast	6	6	66.7	33.3	0.0	0.0
Tumors of the digestive system	Adenomatous polyp, low-grade dysplasia	50	14	71.4	21.4	7.1	0.0
	Adenomatous polyp, high-grade dysplasia	50	26	76.9	11.5	11.5	0.0
	Adenocarcinoma of the colon	2482	1817	97.5	2.3	0.1	0.1
	Gastric adenocarcinoma, diffuse type	215	196	95.9	3.1	1.0	0.0
	Gastric adenocarcinoma, intestinal type	215	200	89.0	5.0	6.0	0.0
	Gastric adenocarcinoma, mixed type	62	59	94.9	1.7	1.7	1.7
	Adenocarcinoma of the esophagus	83	73	84.9	8.2	5.5	1.4
	Squamous cell carcinoma of the esophagus	76	63	90.5	7.9	1.6	0.0
	Squamous cell carcinoma of the anal canal	91	55	74.5	21.8	3.6	0.0
	Cholangiocarcinoma	233	174	70.7	17.8	6.3	5.2
	Gallbladder adenocarcinoma	51	45	73.3	15.6	8.9	2.2
	Gallbladder Klatskin tumor	42	33	87.9	9.1	0.0	3.0
	Hepatocellular carcinoma	201	180	96.7	2.2	0.6	0.6
	Ductal adenocarcinoma of the pancreas	659	509	86.8	9.4	2.9	0.8
	Pancreatic/Ampullary adenocarcinoma	98	78	87.2	10.3	1.3	1.3
	Acinar cell carcinoma of the pancreas	18	16	87.5	12.5	0.0	0.0
	Gastrointestinal stromal tumor (GIST)	62	48	6.3	4.2	0.0	89.6
	Appendix, neuroendocrine tumor (NET)	25	10	90.0	0.0	10.0	0.0
	Colorectal, neuroendocrine tumor (NET)	12	5	100.0	0.0	0.0	0.0
	Ileum, neuroendocrine tumor (NET)	53	35	88.6	8.6	2.9	0.0
	Pancreas, neuroendocrine tumor (NET)	101	65	90.8	6.2	3.1	0.0
	Colorectal, neuroendocrine carcinoma (NEC)	14	4	75.0	25.0	0.0	0.0
	Ileum, neuroendocrine carcinoma (NEC)	8	4	100.0	0.0	0.0	0.0
	Gallbladder, neuroendocrine carcinoma (NEC)	4	2	50.0	0.0	50.0	0.0
	Pancreas, neuroendocrine carcinoma (NEC)	14	12	91.7	8.3	0.0	0.0
Tumors of the urinary system	Non-invasive papillary urothelial carcinoma, pTa G2 low grade	87	80	100.0	0.0	0.0	0.0
	Non-invasive papillary urothelial carcinoma, pTa G2 high grade	80	74	98.6	1.4	0.0	0.0
	Non-invasive papillary urothelial carcinoma, pTa G3	126	115	93.0	0.9	6.1	0.0
	Urothelial carcinoma, pT2-4 G3	416	342	83.3	6.4	10.2	0.0
	Squamous cell carcinoma of the bladder	22	21	90.5	9.5	0.0	0.0
	Small cell neuroendocrine carcinoma of the bladder	4	4	75.0	25.0	0.0	0.0
	Sarcomatoid urothelial carcinoma	23	19	73.7	10.5	0.0	15.8
	Urothelial carcinoma of the kidney pelvis	62	56	85.7	12.5	0.0	1.8
	Clear cell renal cell carcinoma	1730	1397	99.6	0.3	0.1	0.0
	Papillary renal cell carcinoma	513	403	98.3	1.0	0.5	0.2
	Clear cell (tubulo) papillary renal cell carcinoma	26	20	100.0	0.0	0.0	0.0
	Chromophobe renal cell carcinoma	207	169	99.4	0.6	0.0	0.0
	Oncocytoma of the kidney	247	200	97.5	2.5	0.0	0.0
Tumors of the male genital organs	Adenocarcinoma of the prostate, Gleason 3 + 3	83	74	89.2	1.4	9.5	0.0
	Adenocarcinoma of the prostate, Gleason 4 + 4	80	67	68.7	17.9	13.4	0.0
	Adenocarcinoma of the prostate, Gleason 5 + 5	85	79	84.8	6.3	8.9	0.0
	Adenocarcinoma of the prostate (recurrence)	258	188	98.9	1.1	0.0	0.0
	Small cell neuroendocrine carcinoma of the prostate	2	1	100.0	0.0	0.0	0.0
	Seminoma	680	533	94.2	5.4	0.4	0.0
	Embryonal carcinoma of the testis	43	38	73.7	18.4	2.6	5.3
	Leydig cell tumor of the testis	31	31	100.0	0.0	0.0	0.0
	Sertoli cell tumor of the testis	2	2	100.0	0.0	0.0	0.0
	Sex cord stromal tumor of the testis	1	1	100.0	0.0	0.0	0.0
	Spermatocytic tumor of the testis	1	1	100.0	0.0	0.0	0.0
	Yolk sac tumor	51	44	65.9	27.3	6.8	0.0
	Teratoma	52	42	88.1	9.5	2.4	0.0
	Squamous cell carcinoma of the penis	92	72	80.6	19.4	0.0	0.0
Tumors of endocrine organs	Adenoma of the thyroid gland	63	56	98.2	0.0	1.8	0.0
	Papillary thyroid carcinoma	341	300	96.3	3.7	0.0	0.0
	Follicular thyroid carcinoma	109	90	98.9	1.1	0.0	0.0
	Medullary thyroid carcinoma	57	51	96.1	3.9	0.0	0.0
	Parathyroid gland adenoma	43	42	100.0	0.0	0.0	0.0
	Anaplastic thyroid carcinoma	19	17	23.5	47.1	23.5	5.9
	Adrenal cortical adenoma	42	41	92.7	7.3	0.0	0.0
	Adrenal cortical carcinoma	27	17	82.4	5.9	11.8	0.0
	Pheochromocytoma	51	43	30.2	11.6	58.1	0.0
Tumors of hematopoetic and lymphoid tissues	Hodgkin’s lymphoma	103	56	98.2	0.0	0.0	1.8
	Small lymphocytic lymphoma, B-cell type (B-SLL/B-CLL)	50	38	100.0	0.0	0.0	0.0
	Diffuse large B cell lymphoma (DLBCL)	113	99	100.0	0.0	0.0	0.0
	Follicular lymphoma	88	77	94.8	5.2	0.0	0.0
	T-cell non-Hodgkin’s lymphoma	25	18	100.0	0.0	0.0	0.0
	Mantle cell lymphoma	18	14	100.0	0.0	0.0	0.0
	Marginal zone lymphoma	16	13	100.0	0.0	0.0	0.0
	Diffuse large B-cell lymphoma (DLBCL) in the testis	16	14	100.0	0.0	0.0	0.0
	Burkitt lymphoma	5	1	100.0	0.0	0.0	0.0
Tumors of soft tissue and bone	Granular cell tumor	19	16	6.3	6.3	31.3	56.3
	Leiomyoma	50	40	60.0	22.5	15.0	2.5
	Leiomyosarcoma	92	74	54.1	23.0	9.5	13.5
	Liposarcoma	92	85	72.9	21.2	3.5	2.4
	Malignant peripheral nerve sheath tumor (MPNST)	14	14	42.9	35.7	0.0	21.4
	Myofibrosarcoma	25	25	64.0	24.0	8.0	4.0
	Angiosarcoma	34	30	26.7	10.0	3.3	60.0
	Angiomyolipoma	85	82	75.6	14.6	9.8	0.0
	Dermatofibrosarcoma protuberans	20	16	37.5	18.8	6.3	37.5
	Ganglioneuroma	13	13	76.9	15.4	7.7	0.0
	Kaposi sarcoma	6	4	25.0	25.0	0.0	50.0
	Neurofibroma	100	92	70.7	15.2	5.4	8.7
	Sarcoma, not otherwise specified (NOS)	68	64	50.0	26.6	7.8	15.6
	Paraganglioma	40	37	97.3	0.0	2.7	0.0
	Ewing sarcoma	16	15	86.7	0.0	6.7	6.7
	Rhabdomyosarcoma	7	6	50.0	16.7	0.0	33.3
	Schwannoma	56	51	7.8	29.4	23.5	39.2
	Synovial sarcoma	11	10	40.0	40.0	20.0	0.0
	Osteosarcoma	17	14	50.0	21.4	0.0	28.6
	Chondrosarcoma	15	11	81.8	0.0	18.2	0.0
	Rhabdoid tumor	5	4	25.0	50.0	0.0	25.0

**Table 2:** Nestin immunostaining and tumor phenotype

			Nestin immunostaining				
		n	negative (%)	weak (%)	moderate (%)	strong (%)	P
Invasive breast carcinoma of no special type	pT1	502	96.2	2.8	0.4	0.6	0.0103
	pT2	402	93.5	4.2	1.5	0.7	
	pT3-4	81	93.8	1.2	4.9	0.0	
	G1	90	100.0	0.0	0.0	0.0	< 0.0001
	G2	611	96.9	2.6	0.3	0.2	
	G3	316	87.7	6.6	3.5	2.2	
	pN0	214	93.9	2.3	1.9	1.9	0.0932
	pN+	178	93.3	5.6	1.1	0.0	
	HER2 negative	285	94.0	4.6	0.7	0.7	0.8595
	HER2 positive	49	93.9	4.1	2.0	0.0	
	ER negative	27	77.8	11.1	3.7	7.4	0.0054
	ER postive	310	95.5	3.9	0.6	0.0	
	PR negative	81	85.2	9.9	2.5	2.5	0.0051
	PR postive	253	96.8	2.8	0.4	0.0	
	non-triple negative	308	95.1	3.9	1.0	0.0	0.0028
	triple negative	19	73.7	15.8	0.0	10.5	
Clear cell renal cell carcinoma	ISUP 1	277	100.0	0.0	0.0	0.0	0.0149
	ISUP 2	552	99.8	0.2	0.0	0.0	
	ISUP 3	298	99.7	0.3	0.0	0.0	
	ISUP 4	83	97.6	0.0	2.4	0.0	
	Fuhrman 1	55	100.0	0.0	0.0	0.0	0.0006
	Fuhrman 2	609	100.0	0.0	0.0	0.0	
	Fuhrman 3	245	100.0	0.0	0.0	0.0	
	Fuhrman 4	87	95.4	2.3	2.3	0.0	
	Thoenes 1	315	100.0	0.0	0.0	0.0	0.0099
	Thoenes 2	409	100.0	0.0	0.0	0.0	
	Thoenes 3	90	96.7	2.2	1.1	0.0	
	UICC 1	289	100.0	0.0	0.0	0.0	1.0000
	UICC 2	34	100.0	0.0	0.0	0.0	
	UICC 3	73	100.0	0.0	0.0	0.0	
	UICC 4	61	100.0	0.0	0.0	0.0	
	pT1	816	99.9	0.1	0.0	0.0	0.1266
	pT2	138	100.0	0.0	0.0	0.0	
	pT3-4	384	99.0	0.5	0.5	0.0	
	pN0	317	100.0	0.0	0.0	0.0	0.3076
	pN+	32	96.9	3.1	0.0	0.0	
	M0	94	100.0	0.0	0.0	0.0	1.0000
	M1	79	100.0	0.0	0.0	0.0	
Papillary renal cell carcinoma	ISUP 1	41	100.0	0.0	0.0	0.0	0.0101
	ISUP 2	198	100.0	0.0	0.0	0.0	
	ISUP 3	87	97.7	1.1	1.1	0.0	
	ISUP 4	7	85.7	0.0	0.0	14.3	
	Fuhrman 1	1	100.0	0.0	0.0	0.0	0.0230
	Fuhrman 2	159	100.0	0.0	0.0	0.0	
	Fuhrman 3	72	97.2	1.4	1.4	0.0	
	Fuhrman 4	10	90.0	0.0	0.0	10.0	
	Thoenes 1	49	100.0	0.0	0.0	0.0	0.9400
	Thoenes 2	135	99.3	0.0	0.7	0.0	
	Thoenes 3	16	100.0	0.0	0.0	0.0	
	UICC 1	90	98.9	0.0	1.1	0.0	0.9673
	UICC 2	13	100.0	0.0	0.0	0.0	
	UICC 3	5	100.0	0.0	0.0	0.0	
	UICC 4	11	100.0	0.0	0.0	0.0	
	pT1	259	99.6	0.0	0.4	0.0	0.0649
	pT2	53	100.0	0.0	0.0	0.0	
	pT3-4	47	95.7	2.1	0.0	2.1	
	pN0	78	100.0	0.0	0.0	0.0	0.5083
	pN+	19	94.7	5.3	0.0	0.0	
	M0	26	100.0	0.0	0.0	0.0	1.0000
	M1	11	100.0	0.0	0.0	0.0	
Urothelial bladder carcinoma	pTa G2 low	340	99.7	0.3	0.0	0.0	0.9062
	pTa G2 high	149	99.3	0.7	0.0	0.0	
	pTa G3	92	100.0	0.0	0.0	0.0	
	pT2	278	89.2	4.7	5.0	1.1	0.0918
	pT3	421	91.0	6.2	2.6	0.2	
	pT4	203	89.2	6.9	2.5	1.5	
	G2	99	92.9	3.0	4.0	0.0	0.7029*
	G3	1178	91.2	4.8	3.3	0.7	
	pN0	452	89.8	7.1	2.7	0.4	0.6157*
	pN+	309	91.6	4.5	3.6	0.3	
Adenocarcinoma of the pancreas	pT1	8	100.0	0.0	0.0	0.0	0.1229
	pT2	63	93.7	4.8	1.6	0.0	
	pT3	313	87.9	9.6	1.6	1.0	
	pT4	26	84.6	11.5	0.0	3.8	
	G1	14	92.9	0.0	7.1	0.0	0.2460
	G2	289	88.9	9.0	1.4	0.7	
	G3	89	91.0	7.9	1.1	0.0	
	pN0	86	86.0	9.3	3.5	1.2	0.5825
	pN+	324	89.8	8.6	0.9	0.6	
	R0	215	89.3	7.9	2.3	0.5	0.5308
	R1	169	88.2	10.1	0.6	1.2	
	MMR proficient	379	88.1	9.2	1.6	1.1	0.9700
	MMR deficient	2	100.0	0.0	0.0	0.0	
Adenocarcinoma of the stomach	pT1-2	57	96.5	3.5	0.0	0.0	0.5991
	pT3	123	93.5	5.7	0.8	0.0	
	pT4	121	96.7	3.3	0.0	0.0	
	pN0	77	93.5	6.5	0.0	0.0	0.7970
	pN+	224	96.0	3.6	0.4	0.0	
	MMR proficient	252	94.4	4.8	0.4	0.4	0.8955
	MMR deficient	39	92.3	7.7	0.0	0.0	
Endometrioid endometrial carcinoma	pT1	90	72.2	24.4	2.2	1.1	0.1686
	pT2	23	56.5	26.1	13.0	4.3	
	pT3-4	32	71.9	25.0	3.1	0.0	
	pN0	44	70.5	25.0	2.3	2.3	0.7192
	pN+	27	77.8	22.2	0.0	0.0	
Ovarian serous carcinoma, high grade	pT1	21	81.0	19.0	0.0	0.0	0.0477
	pT2	29	72.4	20.7	0.0	6.9	
	pT3	178	75.3	23.6	1.1	0.0	
	pN0	56	73.2	23.2	1.8	1.8	0.2608
	pN1	115	81.7	18.3	0.0	0.0	
Adenocarcinoma of the colon	pT1	75	97.3	2.7	0.0	0.0	0.1154
	pT2	428	98.1	1.9	0.0	0.0	
	pT3	1171	96.5	3.2	0.2	0.1	
	pT4	410	95.4	4.1	0.2	0.2	
	pN0	1089	97.6	2.3	0.1	0.0	0.0682
	pN+	989	95.6	4.0	0.2	0.2	
	V0	1478	96.8	3.1	0.1	0.0	0.0970
	V1	513	95.7	3.5	0.4	0.4	
	L0	750	96.8	3.2	0.0	0.0	0.3179
	L1	1250	96.5	3.1	0.2	0.2	
	right side	356	98.3	1.7	0.0	0.0	0.8185
	left side	1010	97.6	2.2	0.1	0.1	
	MMR proficient	1206	96.1	3.6	0.2	0.2	0.9438
	MMR deficient	118	96.6	3.4	0.0	0.0	
	RAS wildtype	529	96.6	3.0	0.2	0.2	0.2762
	RAS mutation	414	93.5	6.0	0.2	0.2	
	BRAF wildtype	301	92.4	7.0	0.3	0.3	0.5498
	BRAF V600E mutation	51	86.3	11.8	2.0	0.0	

*only in pT2-4 urothelial bladder carcinomas, abbreviation: pT: pathological tumor stage, G: Grade, pN: pathological lymph node status, pM: pathological status of distant metastasis, MMR: mismatch repair, ISUP: International Society of Urological Pathology, UICC: Union for International Cancer Control

## Discussion

**Fig. 4:**
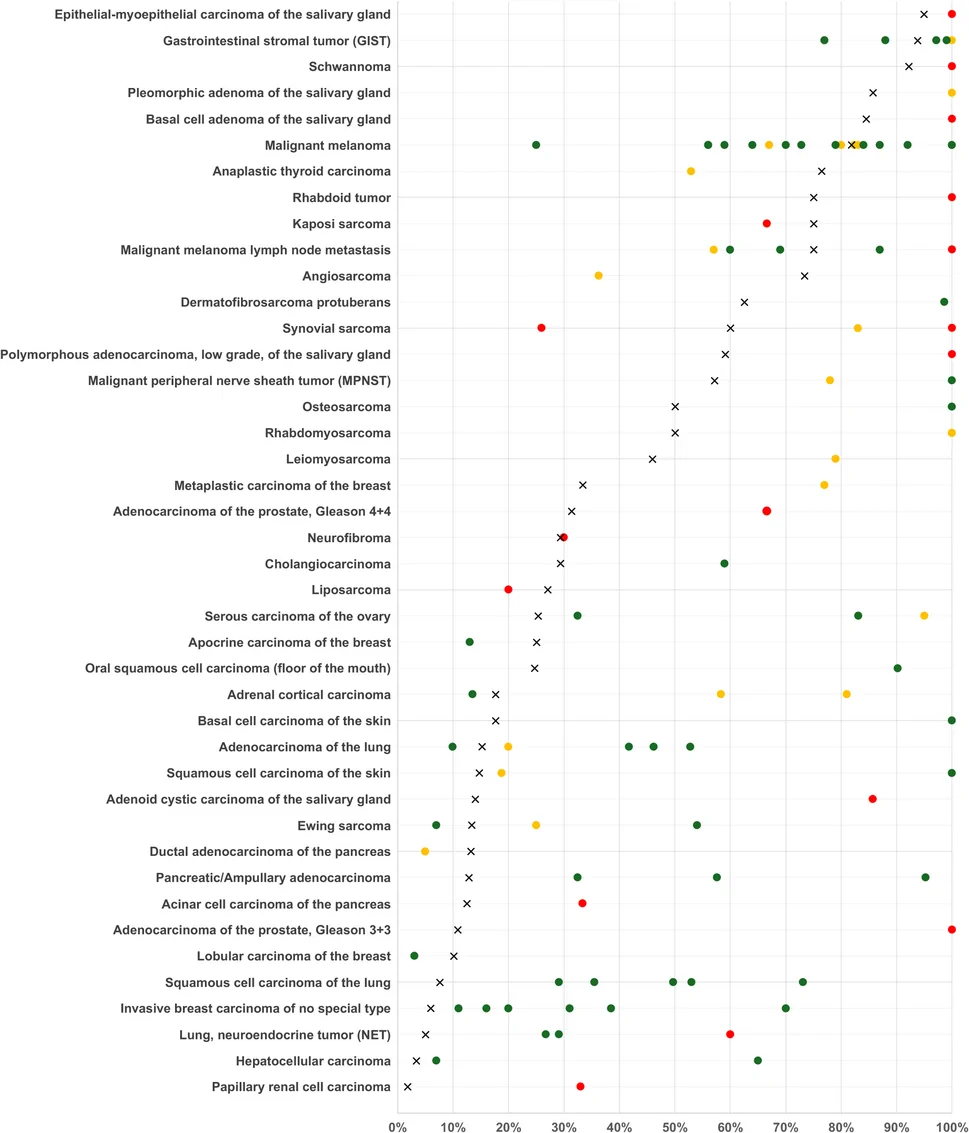
Nestin protein expression in cancer (own findings vs. literature data). Graphical representation of nestin data from this study (X) compared to the previous literature. The colors of the dots represent the number of tumors analyzed in these studies: red: *n* ≤ 20; yellow: *n* = 21 to 100; green: *n* > 100. For raw data and references, see suppl. Table 1

The data from our study, in which 13,318 tumors from 148 different tumor entities were successfully analyzed, provide a comprehensive overview of nestin expression in human tumors. In line with its rather rare expression in normal epithelial cells, nestin expression predominated in neuroectodermal, mesenchymal, and myoepithelial tumors. Tumors with the highest nestin positivity rate included melanomas (our study: 81.8%), schwannomas (92.2%), rhabdoid tumors (75%), angiosarcomas (73.3%), GISTs (93.8%), and tumors of the salivary glands with myoepithelial differentiation such as pleomorphic (100%) and basal cell adenomas (84.5%), as well as myoepitheliomas (100%). These data are largely consistent with nestin positivity rates described in previous IHC studies (summarized in Fig. 4). For example, nestin positivity had been described in 82.4% of 17 [22] and 87.0% of 40 [49] malignant melanomas, 100% of 11 pleomorphic and 100% of 7 basal cell adenomas of the salivary gland [50], 99.0% of 177 [51] and 97.2% of 72 GIST [52], and in 100% of 10 schwannomas [53].

A ranking order of cancers according to their rate of nestin positivity is a major result of our study. It is of note that such a list could not have been compiled from literature data because many tumor types had previously not been analyzed and many literature findings were inconsistent or could not been confirmed in our set of data. For example, in a TMA comprising a total of 208 pheochromocytomas (PCC) and paragangliomas (PGL), an overall nestin positivity rate of 3.0% [54] was reported, others have demonstrated a nestin positivity in 10.5% of 38 PGLs [55] (our findings: 69.8% of PCCs positive / 2.7% of PGLs positive), and very high positivity rates of > 95% [34,53,56, 57] have been found in basal cell carcinoma of the skin (our data 17.6%), osteosarcoma (50%), MPNST (57.1%), or synovial sarcoma (60%). Altogether, the prevalence of nestin positivity varied markedly between tumor entities. As about 15% of entities had > 60% of positive cases and around 50% of tumor types had < 20% of positive cases, nestin IHC could potentially assist in the distinction of tumor types. However, as the level of nestin expression was often rather similar in tumors that are difficult to distinguish morphologically such as - for example - different types of sarcomas, the number of potential diagnostic applications is limited. The markedly higher rate of nestin positivity in gastrointestinal stroma tumors (GIST, 93.8%) than in leiomyomas (40%) and in leiomyosarcomas (45.9%), in adrenocortical carcinoma (17.6%) than in adrenocortical adenomas (7.3%), and in pheochromocytoma (69.8%) than in adrenocortical neoplasms (7.3–17.6%) could - in principle - be exploited for diagnostic purposes. Similarly, nestin IHC positivity could be viewed as an argument for a metastatic liver cancer or a cholangiocellular carcinoma as hepatocellular carcinomas are only rarely (3.3%) nestin positive. That nestin expression analysis will indeed become a diagnostic tool appears unlikely, however, because better markers exist for these differential diagnoses.

The availability of sufficiently large tumor cohorts enabled us to analyze the relationship between nestin expression and histopathological parameters of cancer aggressiveness in 9 different tumor entities. That high nestin expression was linked to unfavorable tumor features in 5 of those tumors suggests that nestin expression may be generally linked to cancer progression and increased tumor malignancy. The relationship with aggressive disease was particularly evident in invasive breast cancer NST. At least 8 studies had previously analyzed the role of nestin in breast cancer and three of them had also found associations with higher histological grade, larger tumor size [24], distant metastasis [26], and/or triple negative phenotype [25]. Other studies examining nestin expression by IHC have reported associations between high nestin expression levels and increased cancer aggressiveness in melanoma [20–22], lung [23], breast[24–27], and pancreatic cancer [28].

Many previous studies using cell lines and animal models have provided evidence for multiple mechanisms that may drive aggressive behavior of nestin expressing cancer cells. For example, earlier studies have found a link between nestin expression and cancer-associated cell properties such as migration and invasion [11,16,58–60], epithelial-mesenchymal transition (EMT) [16,61], proliferation [11,16], angiogenesis [17–19] and stemness [7,11]. At a molecular level nestin can influence tumor cell behavior by directly interacting with proto-oncogenic signaling pathways such as TGF-ß/SMAD [13], Hedgehog (Hh) [14], Akt/GSK3β-Rb [36], and Wnt/β-catenin [16]. For example, nestin regulates EMT and metastasis in pancreatic carcinoma through the interaction with the TGF-ß1/Smad4 signaling [13] and promotes malignant proliferation in an NSCLC cell line by activating the Akt/GSK3β/Rb pathway [36]. In breast cancer stem cells, nestin silencing reduced proliferation by inhibiting WNT/ß-catenin signaling and induced cell cycle arrest and apoptosis, while downregulating the expression of EMT related genes [16]. Nestin is strongly linked to cancer stem cell biology and is regarded as an established stem cell marker in numerous tumor entities [62]. Nestin-positive tumor cells have an increased ability to form spheres, indicating an increased self-renewal capacity [62]. This association is particularly pronounced in pancreatic ductal adenocarcinoma, where nestin is considered a central mediator of the self-renewal capacity of sarcomatoid cancer cell variants and is closely correlated with metastasis [63].

Additional relevance of nestin expression in cancer may be derived from its potential suitability as a therapeutic target. For example, Yamahatsu et al. [17] were able to inhibit tumor angiogenesis by small interfering RNA (siRNA) targeting nestin transcripts in pancreatic cancer. The siRNA administration inhibited growth of vascular endothelial cell lines and inhibited pancreatic cancer cell growth in nude mice [17]. Matsuda et al. [64] also reported inhibition of growth, migration, invasion and sphere-forming ability by siRNA in pancreatic cell lines. In line with these observations, Su et al. [13] inhibited nestin expression by various anti-microtubule inhibitors, such as Aferin A and Cytochalasin D, leading to the inhibition of cell invasion in pancreatic cancer cells.

Given the large scale of the study, the assay used in this study was extensively validated by comparing our IHC findings in normal tissues with data obtained by another independent anti-nestin antibody and RNA data derived from three different publicly accessible databases [65–68]. This approach followed the recommendations of the International Working Group for Antibody Validation [69]. The identification of cross-reactive antibody binding to other proteins than the target protein represents the most significant challenge for antibody validation which is hard to achieve in cell line experiments. As the tissues from 76 different normal tissue categories (8 donors each) will contain most cell types of adult humans, it is very likely that a near-complete spectrum of human proteins with a broad range of posttranslational modifications was evaluated for potential cross-reactivities in our study. The validity of our assay was supported by the highest nestin RNA levels in tissues with highest density of nestin positive endothelial cells such as the heart, skeletal muscle, and the spleen and even more by the confirmation of all positively stained structures seen with our assay by the validation antibody SP103. RNA-based analyses are often challenging, however, as expression measurements do not allow for unequivocal assignment to specific cell types and therefore cannot adequately reflect cell-type-specific protein expression. The numerous additional membranous and cytoplasmic staining patterns seen only by SP103 were considered antibody specific cross-reactivities of this antibody. As SP103 was used in more than 110 publications according to CiteAB [70], these cross-reactivities may have contributed to the high numbers of nestin positive cases for many different tumor types in the literature.

Torhorst et al. previously compared whole sections and TMAs in terms of their suitability for detecting prognostic parameters in a cohort of 553 breast carcinomas. They demonstrated that a single tumor sample in a TMA is sufficient to accurately identify clinical associations. TMAs showed an association with tumor-specific survival that was at least as significant, and in some cases even more significant than, those observed in whole-section analyses [71]. A whole section with a thickness of 2.5 μm, containing a tumor area of 3 × 2 cm, accounts for only about 0.0023% of a tumor mass with a diameter of 5 cm. Furthermore, the amount of tumor tissue analyzed per patient varies widely with whole sections, ranging from < 1 mm² to > 6 cm². TMAs consistently provide standardized representativeness of IHC data for overall tumor masses [44,71]. Multiple samples per tumor within the TMAs would require considerably more workload and significantly increase the amount of tissue required, without substantially improving the representativeness of the whole tumor [44]. However, our study has certain limitations. Although a wide variety of different tumor types and subtypes were analyzed, the number of samples was not always as large as would have been desirable. This applies in particular to some rare tumor types, which in our TMA are represented only in small numbers or not at all. Furthermore, clinical data are lacking for some tumor entities to investigate the clinical significance of Nestin in greater detail.

In summary, the data from this study provide an overview of nestin expression in human cancer and demonstrate that increased nestin levels can occur in many different tumor entities. Significant associations with unfavorable tumor characteristics in multiple entities argue for a role of nestin in increasing cancer aggressiveness. Early experimental data from previous literature on its potential suitability as a therapeutic target in aggressive tumor types such as pancreatic cancer could further support the concept of exploring nestin as a target molecule for drugs.

## Supplementary information

Supplementary Material 1

Supplementary Material 2

Supplementary Material 3

Supplementary Material 4

## Data Availability

All data generated or analyzed during this study are included in this published article.

## Abbreviations

IHC: Immunohistochemistry
TMA: Tissue Microarray
GIST: Gastrointestinal Stromal Tumor
ER: Estrogen Receptor
PR: Progesterone Receptor
NST: No special type
ISUP: International Society of urological Pathology
