# Supplementary material for: Expression of nestin in cancer: a tissue microarray study involving 13,318 cancers from 148 tumor entities
Source: BMC Cancer. 2026 Nov 2;26:1340. doi: 10.1186/s12885-026-17190-w (PMC13729484; doi:10.1186/s12885-026-17190-w)
Supplement: Supplementary file 1 [file 12885_2026_17190_MOESM2_ESM.pdf]

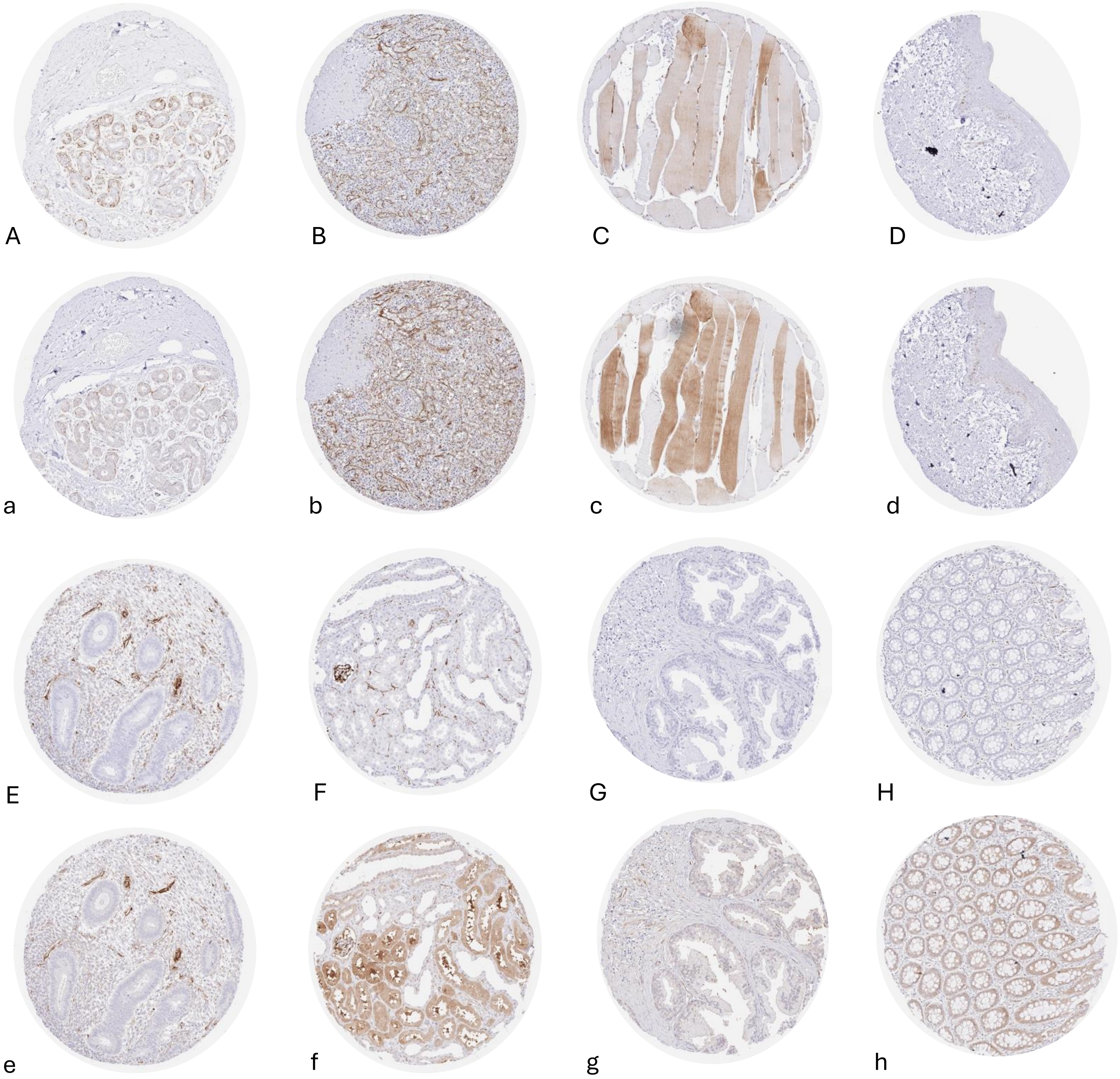
A
B
C
D
a
b
c
d
E
F
G
H
e
f
g
h
