# Supplementary material for: Expression of nestin in cancer: a tissue microarray study involving 13,318 cancers from 148 tumor entities
Source: BMC Cancer. 2026 Nov 2;26:1340. doi: 10.1186/s12885-026-17190-w (PMC13729484; doi:10.1186/s12885-026-17190-w)
Supplement: Supplementary file 2 [file 12885_2026_17190_MOESM3_ESM.pdf]

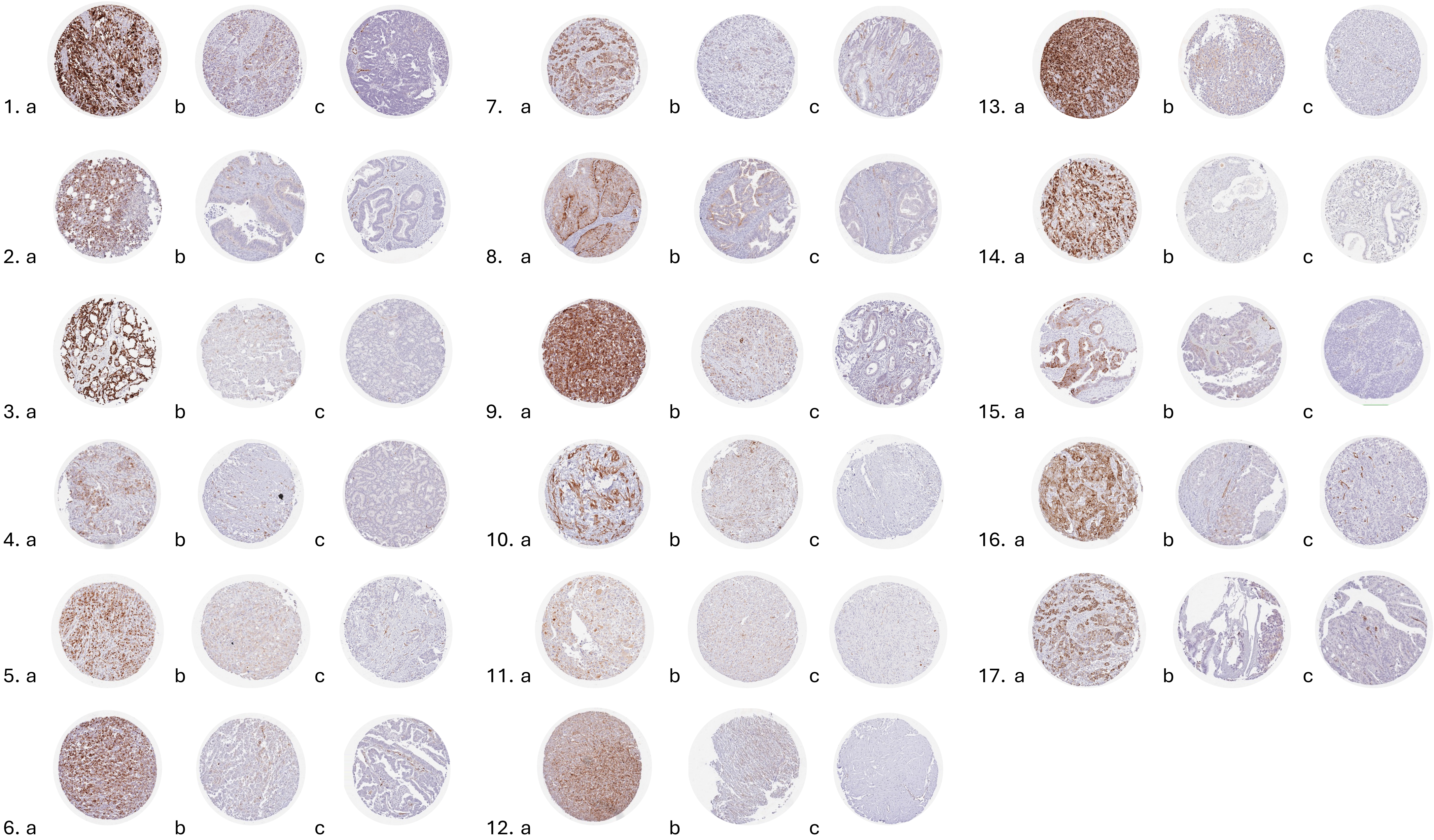
1. a
b
c
7. a
b
c
13. a
b
c
2. a
b
c
8. a
b
c
14. a
b
c
3. a
b
c
9. a
b
c
15. a
b
c
4. a
b
c
10. a
b
c
16. a
b
c
5. a
b
c
11. a
b
c
17. a
b
c
6. a
b
c
12. a
b
c
