# Supplementary material for: Expression of nestin in cancer: a tissue microarray study involving 13,318 cancers from 148 tumor entities
Source: BMC Cancer. 2026 Nov 2;26:1340. doi: 10.1186/s12885-026-17190-w (PMC13729484; doi:10.1186/s12885-026-17190-w)
Supplement: Supplementary file 3 [file 12885_2026_17190_MOESM1_ESM.docx]

**Supplementary Table 1. List of raw data and references used to create Figure 4.**

| **Publication** | **PMID** | **Tumor entitiy** | **Number of tumors (n)** | **Positivity (%)** |
| --- | --- | --- | --- | --- |
| Florenes et al. 1994 | 8275467 | Malignant melanoma | 15 | 67% |
| Ehrmann et al. 2005 | 15677549 | Malignant melanoma | 9 | 100% |
| Klein et al. 2007 | 17143262 | Malignant melanoma | 63 | 70% |
| Brychtova et al. 2007 | 17448190 | Malignant melanoma | 42 | 84% |
| Mihic-Probst et al. 2007 | 17597110 | Malignant melanoma | 56 | 25% |
| Piras et al. 2009 | 19956860 | Malignant melanoma | 130 | 64% |
| Tanabe et al. 2010 | 20156773 | Malignant melanoma | 78 | 59% |
| Kanoh et al. 2010 | 20536663 | Malignant melanoma | 15 | 80% |
| Bakos et al. 2010 | 19845757 | Malignant melanoma | 57 | 56% |
| Sharma et al. 2010 | 20638684 | Malignant melanoma | 40 | 87% |
| Laga et al. 2011 | 21410764 | Malignant melanoma | 105 | 79% |
| Miller et al. 2012 | 22157936 | Malignant melanoma | 23 | 83% |
| Lai et al. 2013 | 23072594 | Malignant melanoma | 64 | 72% |
| Akiyama et al. 2013 | 23417116 | Malignant melanoma | 17 | 82% |
| Ladstein et al. 2014 | 24030749 | Malignant melanoma | 348 | 92% |
| Murtas et al. 2014 | 25034654 | Malignant melanoma | 75 | 56% |
| Sabet et al. 2014 | 25339000 | Malignant melanoma | 12 | 100% |
| Plaza et al. 2016 | 26661921 | Malignant melanoma | 40 | 100% |
| Olsen et al. 2006 | 16528378 | Ewing sarcoma | 27 | 7% |
| Murphy et al. 2009 | 19417621 | Ewing sarcoma | 16 | 25% |
| Sadikovic et al. 2011 | 21162641 | Ewing sarcoma | 28 | 54% |
| Shimada et al. 2007 | 17300669 | Liposarcoma | 10 | 20% |
| Shimada et al. 2007 | 17300669 | Schwannoma | 10 | 100% |
| Shimada et al. 2007 | 17300669 | Leiomyosarcoma | 19 | 79% |
| Shimada et al. 2007 | 17300669 | Neurofibroma | 10 | 30% |
| Kleeberger et al. 2007 | 17909025 | Adenocarcinoma of the prostate, Gleason 3+3 | 4 | 100% |
| Kleeberger et al. 2007 | 17909025 | Adenocarcinoma of the prostate, Gleason 4+4 | 3 | 66% |
| Kanoh et al. 2008 | 18693153 | Squamous cell carcinoma of the skin | 16 | 18% |
| Sabet et al. 2014 | 25339000 | Squamous cell carcinoma of the skin | 31 | 100% |
| Piras et al. 2009 | 19956860 | Malignant melanoma lymph node metastasis | 32 | 69% |
| Bakos et al. 2010 | 19845757 | Malignant melanoma lymph node metastasis | 21 | 57% |
| Sharma et al. 2010 | 20638684 | Malignant melanoma lymph node metastasis | 32 | 87% |
| Fusi et al. 2011 | 20882037 | Malignant melanoma lymph node metastasis | 6 | 100% |
| Murtas et al. 2014 | 25034654 | Malignant melanoma lymph node metastasis | 35 | 60% |
| Chen et al. 2010 | 20132963 | Squamous cell carcinoma of the lung | 26 | 73% |
| Ryuge et al. 2011 | 20829334 | Squamous cell carcinoma of the lung | 31 | 35% |
| Skarda et al. 2012 | 22837134 | Squamous cell carcinoma of the lung | 79 | 29% |
| Narita et al. 2014 | 24481417 | Squamous cell carcinoma of the lung | 74 | 53% |
| Liu et al. 2022 | 36082356 | Squamous cell carcinoma of the lung | 26 | 49% |
| Chen et al. 2010 | 20132963 | Adenocarcinoma of the lung | 25 | 20% |
| Ryuge et al. 2011 | 20829334 | Adenocarcinoma of the lung | 131 | 10% |
| Skarda et al. 2012 | 22837134 | Adenocarcinoma of the lung | 36 | 52% |
| Narita et al. 2014 | 24481417 | Adenocarcinoma of the lung | 48 | 41% |
| Liu et al. 2022 | 36082356 | Adenocarcinoma of the lung | 29 | 46% |
| Murphy et al. 2009 | 19417621 | Rhabdoid tumor | 8 | 100% |
| Gadd et al. 2010 | 20212451 | Rhabdoid tumor | 10 | 100% |
| Shimada et al. 2007 | 17300669 | Rhabdomyosarcoma | 10 | 100% |
| Willoughby et al 2008 | 18528283 | Rhabdomyosarcoma | 12 | 100% |
| Murphy et al. 2009 | 19417621 | Rhabdomyosarcoma | 8 | 100% |
| Sana et al. 2011 | 22156015 | Rhabdomyosarcoma | 7 | 100% |
| Sadikovic et al. 2011 | 21162641 | Rhabdomyosarcoma | 30 | 100% |
| Shimada et al. 2007 | 17300669 | Synovial sarcoma | 8 | 26% |
| Somers et al. 2007 | 17464317 | Synovial sarcoma | 12 | 83% |
| Sadikovic et al. 2011 | 21162641 | Synovial sarcoma | 5 | 100% |
| Ravindran et al. 2015 | 25352468 | Oral squamous cell carcinoma  (floor of the mouth) | 72 | 90% |
| Olsen et al. 2006 | 16528378 | Malignant peripheral nerve sheath tumor (MPNST) | 23 | 78% |
| Shimada et al. 2007 | 17300669 | Malignant peripheral nerve sheath tumor (MPNST) | 35 | 100% |
| Sadikovic et al. 2011 | 21162641 | Malignant peripheral nerve sheath tumor (MPNST) | 5 | 100% |
| Zambo et al. 2012 | 22344865 | Osteosarcoma | 45 | 100% |
| Ryuge et al. 2011 | 20829334 | Lung, neuroendocrine tumor (NET) | 5 | 60% |
| Ryuge et al. 2012 | 22572237 | Lung, neuroendocrine tumor (NET) | 30 | 26% |
| Brominska et al. 2017 | 29517198 | Lung, neuroendocrine tumor (NET) | 48 | 29% |
| Lenz et al. 2011 | 22187705 | Pancreatic/Ampullary adenocarcinoma | 117 | 32% |
| Kim et al. 2012 | 23071857 | Pancreatic/Ampullary adenocarcinoma | 42 | 95% |
| Shan et al. 2015 | 25371063 | Pancreatic/Ampullary adenocarcinoma | 59 | 57% |
| Qin et al. 2012 | 22296500 | Serous carcinoma of the ovary | 123 | 32% |
| He et al. 2014 | 24254558 | Serous carcinoma of the ovary | 113 | 83% |
| Zeng et al. 2014 | 24300467 | Serous carcinoma of the ovary | 20 | 95% |
| Tsujimura et al. 2001 | 11238030 | Gastrointestinal stromal tumor (GIST) | 18 | 100% |
| Sarlomo-Rikala et al. 2002 | 12193211 | Gastrointestinal stromal tumor (GIST) | 67 | 88% |
| Novelli at al. 2010 | 20716168 | Gastrointestinal stromal tumor (GIST) | 177 | 99% |
| Enache et al. 2012 | 22395498 | Gastrointestinal stromal tumor (GIST) | 81 | 77% |
| Peker et al. 2014 | 24755685 | Gastrointestinal stromal tumor (GIST) | 72 | 97% |
| Serra-Guillén et al. 2013 | 23962157 | Dermatofibrosarcoma protuberans | 71 | 98% |
| Yanai et al. 2013 | 24147430 | Pleomorphic adenoma of the salivary gland | 11 | 100% |
| Yanai et al. 2013 | 24147430 | Basal cell adenoma of the salivary gland | 7 | 100% |
| Yanai et al. 2013 | 24147430 | Epithelial-myoepithelial carcinoma of the salivary gland | 2 | 100% |
| Yanai et al. 2013 | 24147430 | Polymorphous adenocarcinoma, low grade, of the salivary gland | 3 | 100% |
| Yanai et al. 2013 | 24147430 | Adenoid cystic carcinoma of the salivary gland | 7 | 85% |
| Sabet et al. 2014 | 25339000 | Basal cell carcinoma of the skin | 55 | 100% |
| Jung et al. 2015 | 25755746 | Anaplastic thyroid carcinoma | 17 | 52% |
| Lan et al. 2015 | 26722452 | Kaposi sarcoma | 3 | 66% |
| Calderaro et al. 2022 | 35987274 | Hepatocellular carcinoma | 313 | 7% |
| Sasaki et al. 2022 | 35076959 | Hepatocellular carcinoma | 75 | 65% |
| Lan et al. 2015 | 26722452 | Angiosarcoma | 22 | 36% |
| Rammal et al. 2024 | 38763422 | Metaplastic carcinoma of the breast | 13 | 77% |
| Calderaro et al. 2022 | 35987274 | Cholangiocarcinoma | 204 | 59% |
| Rammal et al. 2024 | 38763422 | Apocrine carcinoma of the breast | 41 | 13% |
| Toti et al. 2005 | 16136494 | Adrenal cortical carcinoma | 12 | 58% |
| Papathomas et al. 2016 | 27589897 | Adrenal cortical carcinoma | 38 | 13% |
| Lachenmayer et al. 2009 | 19294612 | Adrenal cortical carcinoma | 16 | 81% |
| Ohike et al. 2007 | 17685930 | Ductal adenocarcinoma of the pancreas | 20 | 5% |
| Ohike et al. 2007 | 17685930 | Acinar cell carcinoma of the pancreas | 6 | 33% |
| Piras et al. 2009 | 19956860 | Lobular carcinoma of the breast | 26 | 3% |
| Parry et al. 2008 | 18641405 | Invasive breast carcinoma of no special type | 128 | 11% |
| Liu et al. 2010 | 20028381 | Invasive breast carcinoma of no special type | 131 | 16% |
| Liu et al. 2012 | 22207173 | Invasive breast carcinoma of no special type | 268 | 20% |
| Gao et al. 2014 | 24443256 | Invasive breast carcinoma of no special type | 109 | 38% |
| Nowak et al. 2018 | 29345290 | Invasive breast carcinoma of no special type | 124 | 31% |
| Rammal et al. 2024 | 38763422 | Invasive breast carcinoma of no special type | 183 | 70% |
| Murphy et al. 2009 | 19417621 | Papillary renal cell carcinoma | 6 | 33% |
