# Supplementary material for: Expression of nestin in cancer: a tissue microarray study involving 13,318 cancers from 148 tumor entities
Source: BMC Cancer. 2026 Nov 2;26:1340. doi: 10.1186/s12885-026-17190-w (PMC13729484; doi:10.1186/s12885-026-17190-w)
Supplement: Supplementary file 4 [file 12885_2026_17190_MOESM4_ESM.docx]

**Supplementary Figure 1. Assay validation by comparison of antibodies.** Using ARX-604, a predominantly membranous nestin staining of variable intensity was seen in endothelial and myoepithelial cells of the breast (A), endothelial cells of the spleen (B), endothelial and muscle cells (cytoplasmic) in skeletal muscle (C), endothelial cells of the skin (D), endothelial and stromal cells of the endometrium (E), as well as endothelial cells of the kidney cortex (F), prostate (G) and the colon (H). All these stainings were confirmed by SP103 (a-h), but resulted in significant additional, predominantly cytoplasmic nestin stainings in renal tubular cells (f), in the prostate epithelium (g) and the colon epithelium (h) which were not observed by ARX-604. These additional stainings were considered antibody specific cross-reactivities of SP103. The images A-H and a-h are from consecutive tissue sections.

**Supplementary Figure 2. Examples of nestin immunostaining in cancer.** The panels show examples of a strong (a), weak (b), and negative (c) staining of nestin in a carcinosarcoma of the ovary (1), adenocarcinoma of the colon (2), acinic cell carcinoma of the salivary gland (3), adenocarcinoma of the lung (4), gastric adenocarcinoma, mixed type (5), carcinosarcoma of the uterus (6), adenocarcinoma of the esophagus (7), endometrioid endometrial carcinoma (8), ductal adenocarcinoma of the pancreas (9), schwannoma (10), anaplastic thyroid carcinoma (11), Dermatofibrosarcoma protuberans (12), angiosarcoma (13), adenocarcinoma NOS of the salivary gland (14), serous carcinoma of the ovary (15), invasive breast cancer of no special type (16), and a gastric adenocarcinoma, intestinal type (17).
